# Irregularity and time series trend analysis of rainfall in Johor, Malaysia

**DOI:** 10.1016/j.heliyon.2024.e30324

**Published:** 2024-04-27

**Authors:** Shaidatul Azdawiyah Abdul Talib, Wan Mohd Razi Idris, Liew Ju Neng, Tukimat Lihan, Muhammad Zamir Abdul Rasid

**Affiliations:** a Malaysian Agricultural Research and Development Institute (MARDI), Ibu Pejabat MARDI, Persiaran MARDI-UPM, 43400, Serdang, Selangor, Malaysia; b Department of Earth Sciences and Environment, Faculty of Science & Technology, Universiti Kebangsaan Malaysia, 43600, UKM, Bangi, Selangor, Malaysia; c Center for Water Research and Analysis, Faculty of Science & Technology, Universiti Kebangsaan Malaysia, 43600, UKM, Bangi, Selangor, Malaysia

**Keywords:** Climate, Drought, Flood, Rainfall, Variability

## Abstract

Due to its effect on weather and its propensity to cause catastrophic incidents, climate change has garnered considerable global attention. Depending on the area, the effects of climate change may vary. Rainfall is among the most significant meteorological factors associated with climate change. In Malaysia, changes in rainfall distribution pattern have led to many floods and droughts events which lead to La Nina and El Nino where Johor is one of the states in southern part that usually affected. Thus, rainfall trend analysis is important to identify changes in rainfall pattern as it gives an initial overview for future analysis. This research aims to evaluate historical rainfall data of Johor between 1991 and 2020. Normality and homogeneity tests were used to ensure the quality of data followed by Mann-Kendall and Sen's slope analysis to determine rainfall trend as the rainfall data is not normally distributed (p > 0.05). Standardized precipitation anomaly, coefficient of variation, precipitation concentration index and rainfall anomaly index were used to identify rainfall variability and intensity while standard precipitation index was used to evaluate drought severity. The lowest annual rainfall recorded was 1725.07 mm in 2016 and the highest was 2993.19 mm in 2007. Annual rainfall and seasonal rainfall showed a declining trend although it is not statistically significant (p > 0.05). Results reveal that Johor experienced extreme wet and dry years, leading to drought and flood incidents. Major floods arose in 2006, 2007, 2008, 2010 and 2011 while driest years occurred in 1997, 1998 and 2016 which led to El Nino phenomenon. March and April were identified as the driest months among all. Thus, the findings from this study would assist researchers and decision-makers in the development of applicable adaptation and mitigation strategies to reduce climate change impact. It is recommended that more data analysis from more stations should be done in the future research study to obtain a clearer view and more comprehensive results.

## Graphical abstract

**Figure:**
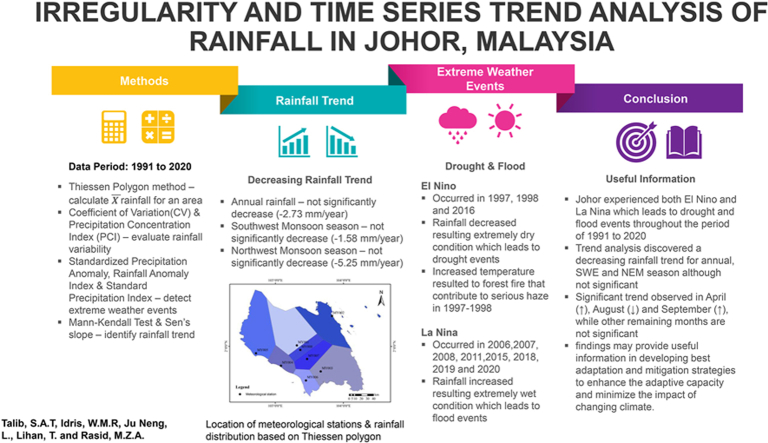

## 1 Introduction

Climate change is presently receiving more attention globally due to its effects on weather and extreme events. Climate change might differ in various aspects on various regional and temporal ranges [1]. Climate change harmfully affects hydrological cycle, affecting rainfall patterns, water supplies, public health, as well as water energy exploitation, and it may also result in numbers of extreme events that can damage the ecosystem [2,3]. Researchers discovered that extreme events occurred more frequently due to the increase of sea level rise and annual temperature, as well as the irregularity of rainfall patterns brought on by changing climate [4]. The increase of sea surface temperature due to changing climate has preceded vital rainfall pattern fluctuations [5]. Rainfall is an essential element in hydrological cycle, and its spatial as well as temporal variability is of major significance considering scientific and socioeconomic prospects [6]. Future changes in rainfall patterns are anticipated to increase and might cause more extreme rainfall events [7].

Corresponding to the Fourth Assessment Report (AR4) by the Intergovernmental Panel on Climate Change (IPCC), in between the year of 1880 and 2012, worldwide ocean and land surface mean temperature has warmed by 0.85 °C, proving that surface warming occurred globally [2]. According to IPCC's Fifth Assessment Report (AR5), between the year 1880 and 2012, global temperature rose around 0.65 °C–1.06 °C, sea level rose to 19 cm, and greenhouse gas (GHG) emissions expected would continue rising [8]. The AR5 reported significant changes of rainfall patterns were observed from 1900 to 2005 in many regions, with rainfall fall decreasing across southern Africa, along the Mediterranean coast, and in some parts of southern Asia [9]. Due to the interrelation of atmosphere, land, and oceans, tropical regions are susceptible to changing climate and abnormality. According to the Southeast Asia Climate Outlook: 2021 Survey Report [10], the region that most vulnerable to climate change is Southeast Asia, and Malaysia is one of the impacted countries. Since the 1960s, for every decade, temperature has been soaring around 0.14 °C–0.20 °C across Southeast Asia, together with an escalating total of warm nights and hot days, along with a plunge in cooler weather [11]. Almost three decades have apparently passed since rainfall abnormalities due to climate change have been reported [12].

It is essential to evaluate how changing climate will influence Malaysia's rainfall patterns. Therefore, various assessments of rainfall have been done to evaluate the changing trends [13]. Earlier studies found that the trend of rainfall frequency, severity, and flood events has changed [14]. Malaysia has experienced several catastrophic events in recent years, including floods and droughts. During monsoon season, floods and flash floods are routinely prevalent along Peninsular Malaysia east coast area [15]. Corresponding to previous research in 2015 [16], in between the year of 1971 and 2010, Peninsular Malaysia experienced a significant annual rainfall increase (95 % confidence level) along with Northeast monsoon (NEM) season rainfall (90 % confidence level). Since 2000, rainfall has increased by 17 % in comparison to 1970 [17]. Severe floods over the east coast of Peninsular Malaysia during 9th to December 11, 2004 was set off by an extreme rainfall incident [18]. Apart from that, cold surges during NEM season led to an abnormal heavy rainfall that struck for several days setting off an enormous flood in the southern part of Peninsular Malaysia during end of December 2006 and middle of January 2007 [19]. Moreover, Madden-Julian Oscillation, Indian Ocean Dipole, and Borneo Vortex also made a substantial contribution to these flood incidents [20].

This demonstrated that rainfall is among the significant climate components that can influence the environment. Changes in rainfall patterns have the potential to cause droughts or floods in different regions. As it relates to a region's water-related issues and associated challenges, rainfall trend information is vital for environmental and water management purposes [21]. In order to design suitable adaptation or mitigation measures in reaction to climate change phenomenon, researchers and decision-makers would benefit from understanding historical and future trends of rainfall patterns. Researchers are likely more interested on the rainfall trend due to the necessity of analyzing hydrological pattern for future flood evaluation and the prevalence of flooding in Malaysia, since one of Malaysia's most frequent natural disasters is flood [22]. The exploitation of Malaysia's natural resources, involving energy development projects, unrestricted pollutant release, huge land restoration, and livestock rearing might perhaps contribute to stream deterioration, erosion, and flood events [22].

Over the years, trend detection methodologies have grown in popularity among academics [23] for climate change study, particularly related to temperature and rainfall pattern [18]. Numerous hydrological and climatic parameter studies employing a variety of methodologies have been conducted. Evaluation of these climatic and hydrological changes is substantially aided by trend analysis [24,25]. In order to reduce uncertainty in trend analysis summaries, complex methods including parametric models (deterministic trend), non-parametric methods, and stochastic trend are utilized [15]. There are numerous statistical methods that have been used to determine rainfall trends, involving parametric and non-parametric tests. It is generally assumed that in statistical parametric tests, processed data are normally distributed [8]. A non-parametric test, conversely, data do not need to be assumed normally distributed [26]. The Mann-Kendall (MK) test is one of the non-parametric tests commonly being used in analysis of hydrological trend as having a minimal sensitivity to abrupt discontinuities in time series besides being robust against outliers and distribution-free [27,28]. The MK test is less susceptible to the outliers and impervious to the true data distribution because it is based on observations levels rather than actual values [29].

Johor located in southern area of Peninsular Malaysia, one of the states that affected by floods and droughts due to changes in rainfall distribution. Intense floods in Malaysia that affected Johor during December 2006 to January 2007, January 2011, December 2015, January 2018, December 2019 to January 2020, and June 2020 was the consequence of a new weather phenomenon brought on by climate change. Typhoon Uthor was the major contributor to severe floods in December 2006 and January 2007 [30] causing 18 deaths, more than 100,000 people were evacuated and about USD 0.5 billion of losses were sustained [31], while the others were caused by tropical cyclone and monsoon season. Flooding events frequently occur in December when the highest rainfall and peak streamflow are recorded [32]. Besides, flash floods are also common to happen in urban areas including Johor Bahru [33]. Flash flood is rapid flooding of geomorphic low-lying areas due to heavy rain pouring on saturated soil or dry soil which is an impermeable area and have poor absorption ability. Apart from that, historical records also indicate several severe drought events in 1990, 1997, 2005, 2010, 2010–2014 and 2019, leading to water supply disruption in Johor [34,35]. Nevertheless, the most intense of several droughts’ incidents happened in Malaysia was during the 1997/1998 El Niño event, which impacted the environment along with all social activities. Persistent dry weather conditions posed a threat of widespread wildfire in a certain spot [36].

Examining the overall rainfall distribution pattern has gained significance in the context of future water resources planning and management for our country. Nevertheless, comprehensive studies on rainfall patterns, particularly in Johor, Malaysia is very limited [37]. In the southern region of the Peninsular Malaysia, where there is no high mountain range separating the west and east regions, the monthly precipitation exhibits a mixture of the rainfall distribution patterns of the west coast and east coast of the Peninsular Malaysia (e.g. Senai) [38]. The former study revealed that there was an increase in the amount of rainfall in Johor Bahru, but the analysis was made for a short period of time such as the study by Ref. [33] or involving outdated (latest at the time of publication) climate normal such as the study by Ref. [39].

Given the significant impacts of rainfall pattern changes on floods, droughts, and overall ecosystem health in Johor, understanding these trends is vital for developing effective climate change adaptation and mitigation strategies. Therefore, the main aim of the present study is to analyze rainfall trend changes and variability in Johor under the new and latest climate normal, 1991 to 2020 [40]. Furthermore, very few studies have been carried out in Johor related to rainfall variability. For that, we investigated the trend in monthly, seasonal, and annual rainfall by applying the Mann–Kendall test and Sen's slope method. Rainfall variability will be determined using coefficient of variation (CV), standardized precipitation anomaly, rainfall anomaly index (RAI), and precipitation concentration index (PCI). Before the trend and variability analysis, the rainfall data will be carefully assessed for homogeneity and normality to ensure the quality of data used. The findings from this study will be helpful for government agencies and other related authorities in developing climate change adaptation and mitigation plans and strategies based on the latest IPCC suggested socio-economic scenarios for better flood and droughts management.

## 2 Study area and methodology

### 2.1 Study area

**Fig. 1:**
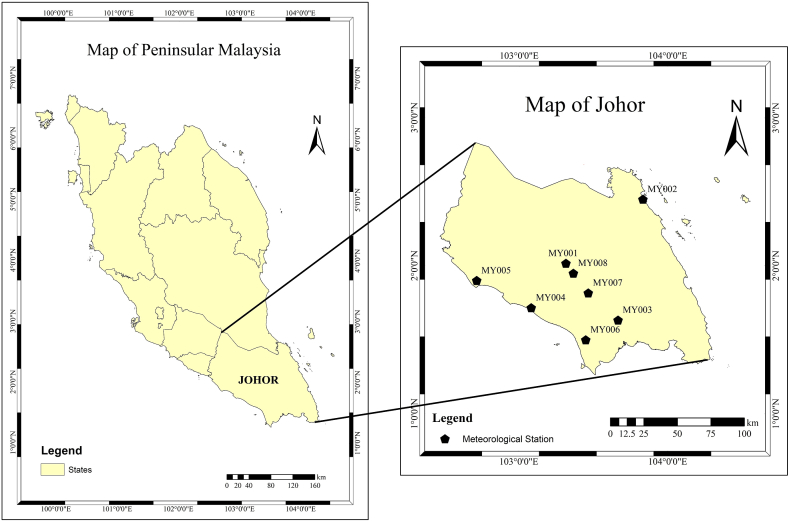
Location of Meteorological stations in Johor.

Johor is one of Malaysia's states, located in Peninsular Malaysia's southern region(1°48′N, 103°76′E) with the total land area of 19,102 km^2^ [39]. According to the Köppen climate classification, Johor has a tropical rainforest climate, characterized by high mean annual temperatures, small temperature variations, and received year-round rainfall [25]. Both monsoonal and convective rainfall systems have a strong correlation with Johor's rainfall. The average annual rainfall is 2600 mm, while daily precipitation ranges from 5 mm to 10 mm. The Southwest Monsoon (SWM) season (May to September), and the NEM (November to March) are two rainy seasons that are distinguished by two relatively short inter-monsoon periods. The NEM creates intense rainfall, specifically to Peninsular Malaysia east coast states, including Johor and western Sarawak, whereas the SWM usually implies comparatively drier weather. Eight stations in Johor were selected for this study (Fig. 1) depending on the data's availability and quality, as shown in Table 1. Calculations, summaries, and analyses of daily data were made using Pivot Table in Microsoft Excel, while statistical analysis was conducted using the XLSTAT-Time Series Analysis module, an add-in of Microsoft Excel.

**Table 1:** Characteristics MetMalaysia meteorological stations.

Station code	Station name	Latitude	Longitude
MY001	Kluang	2.0167°N	103.3167°E
MY002	Mersing	2.4500°N	103.8333°E
MY003	Senai	1.6333°N	103.6667°E
MY004	Pusat Pertanian (PP) Parit Botak	1.7167°N	103.0833°E
MY005	Pusat Pertanian (PP) Sg Sudah	1.9000°N	102.7167°E
MY006	MARDI Pontian	1.5000°N	103.4500°E
MY007	Chemara Layang-Layang	1.8167°N	103.4667°E
MY008	MARDI Kluang	1.9500°N	103.3667°E

### 2.2 Data collection

Required rainfall data were collected from the Meteorological Department of Malaysia (MetMalaysia), at daily time scale from 1974 to 2020. Data from 1974 to 2004 (30 years) were used as the baseline period while data from 1991 to 2020 (30 years) were used to represent the analysis. Application of unreliable observed climate data might cause wrong conclusions on the climate conditions, so data quality control and homogeneity tests were conducted to minimize the error. Data quality control involves removal of stations with inhomogeneous trend, i.e. those with more than 10 % missing and unreasonable values. Incomplete rainfall record is common due to equipment malfunctions and loss of recording, among others. Considering observations from neighboring rainfall stations using thiessen polygon method, incomplete rainfall observations were interpolate using an average station approach. The daily data are then converted to a monthly, annually and seasonally time scale for further analysis. Rainfall data from MetMalaysia is not open to public, where application with justification are required. Therefore, data from NASA Power project which available online (https://power.larc.nasa.gov/data-access-viewer/), and free access to public are suggested as an alternative although real time data collected from weather station on field is the best to represent the selected location.

### 2.3 Homogeneity analysis

The homogeneity test aims to identify and eliminate stations with non-uniform conditions from the trend analysis. Climate data may become inhomogeneous due to variations in environmental factors, instrumentation and measurement approaches which potentially obscuring the actual climate conditions. In this study, four homogeneity tests recommended by previous study by Tan [41] which include Alexandersson's Standard Normal Homogeneity Test (SNHT), Buishand's Range Test (BRT), Pettitt's Test (PT) and Von Neumann's Ratio Test (VNRT) were used to detect the homogenous trend of the monthly, annual and seasonal time series of rainfall at a 5 % significance level. Two hypotheses were used; (i) H_0_ (null hypothesis) – data are homogeneous and (ii) H_a_ (alternative hypothesis) – there is a date at which there is some changes in the data. H_0_ should be rejected when the p-value is lower than the significance level (p < 0.05). Results were then categorized based on the numbers of tests that rejecting H_0_; (i) Class A (Useful) –none or only one the tests rejects H_0_, (ii) Class B (Doubtful) – when two of the tests reject H_0_; and (iii) Class C (Suspect) – when three or all the tests reject H_0_. Analysis were done using the XLSTAT-Time Series Analysis module, an add-in of Microsoft Excel.

### 2.4 Normality test

Normality test is important in order to determine the appropriate methods to evaluate significant trends in precipitation time series data either using parametric or non-parametric trend analysis methods. İn this study, normality test were done using Shapiro-Wilk test to detect the homogenous trend of the monthly, annual and seasonal time series of rainfall at a 5 % significance level. If normality exists in a rainfall series, a parametric test is selected and vice versa [42]. Two hypotheses were used; (i) H_0_ (null hypothesis) – data are normally distributed and (ii) H_a_ (alternative hypothesis) – data is not normally distributed. H_0_ should be rejected when the p-value is lower than the significance level (p < 0.05). Analysis were done using the XLSTAT-Time Series Analysis module, an add-in of Microsoft Excel.

### 2.5 Thiessen polygon

The Thiessen polygon method involves weighting the area ratio of the polygons surrounding the rainfall station to determine the average rainfall for the specified area [43]. For an area within a station, the rainfall from observation stations serves as a proxy for the region's rainfall because it is considered that the rainfall received is comparable to that at the station [44]. The area's rainfall can be approximated using the following equation (Eq. 1):

(1) $$\bar{P}=\sum_{i=1}^{n}A_{i}P_{i}/\sum_{i=1}^{n}A_{i}$$

where, $\bar{P}$ is mean rainfall of the area; $P_{i}\ldots P_{n}$ is rainfall observed at n-stations in the area and $A_{i}\ldots A_{n}$ the areas of each observation point. The Thiessen polygon method computes the average rainfall by weighing each station's area. This method considers both the relative density of the observation network and the placements of the rainfall stations.

Johor areas were divided into polygons with each polygon representing the entire area covered by that polygon (a single point of meteorological station). Analysis was done using ArcGIS 10.8 software where Inverse distance weighting (IDW) technique, a deterministic methodology for multivariate interpolation applying a known set of scattered points was used [45]. Although this is a simple and straightforward method, it has some disadvantages. As an example, the estimation is based on only a single meteorological station and does not incorporate the information on neighboring points. Additionally, there might be sudden jumps or discontinuities across the boundaries of polygons.

### 2.6 Rainfall trend analysis

#### 2.6.1 The Mann Kendall analysis

The MK analysis is a non-parametric test that accepts independent data, takes outliers into account, and is only applied to trustworthy data [[46], [47], [48]]. The MK test is used globally in meteorological parameters trend analysis [46,49] as MK test has been effective when the data is not normally distributed. The MK test does not need assumption of normality as it merely reveals the direction of significant trends, not their magnitude. World Metrological Data has broadly recommended this test for free trend assessment by public [49] besides detecting statistically significant trends of long-term data [46]. In MK test, two hypothesis tests need to be considered to verify the existence of trend in a time series; Null Hypothesis (H_0_) – no trend in the time series and Alternative Hypothesis (H_a_) – time series follow a trend over. In testing hypotheses, the probability value (p-value) is used. If the p-value > α = 0.05, it has failed to reject the H_0_, hence, concluded that there is no trend in the time series. In the other hand, if the p-value < α = 0.05, H_0_ will be rejected, if the time series follow a trend over [50]. Formula below is used for MK tests calculation [51]:

The MK test measured values $\left(x_{j}‐x_{k}\right)$, where j>k;k=1,2,3,….,n‐1;j=2,3,4,….,n and n is the number of data. The test statistics S is computed using the formula as below (Eq. (2)):

(2) $$S=\sum_{k=1}^{n-1}\sum_{j=k+1}^{n}sign\left(x_{j}-x_{k}\right)$$

$sign\left(x_{j}-x_{k}\right)$ is calculated as follows (Eq. (3)):

(3) $$sign\left(x_{j}-x_{k}\right)=\left\{ \begin{array}{c} +1;\left(x_{j}-x_{k}\right)>0\\ 0;\left(x_{j}-x_{k}\right)=0\\ -1;\left(x_{j}-x_{k}\right)<0 \end{array}\right.$$

S is proven to be asymptotically normally distributed based on the following parameters as in Eq. (4).

E(S)=0

(4) $$Var\left(S\right)=\left\{n\left(n-1\right)\left(2n+5\right)-\sum_{p=1}^{g}t_{p}\left(t_{p}-1\right)\left(2t_{p}+5\right)\right\}/18$$

where, g is tied groups quantity in the data set; $t_{p}$ is $p^{th}$ tied group data quantity and n is time series data quantity. A positive value of S implies an increasing trend in the time series while a negative value of S implies the opposite, which indicates a decreasing trend. As for n>10 (observations more than 10), Z (standard normal random variable) can be applied for hypothesis testing (Eq. (5)).

(5) $$Z=\left\{ \begin{array}{c} \left(S-1\right)/{\left[Var\left(S\right)\right]}^{1/2}\;if\;S>0\\ 0\;if\;S=0\\ \left(S+1\right)/{\left[Var\left(S\right)\right]}^{1/2} \end{array}\right.$$

Similar to the value of S, a positive value of Z implies an increasing trend in the time series while a negative value for Z implies the opposite, which means that there is a decreasing trend over the time [17].

#### 2.6.2 Sen's slope (SS) estimator

The SS test is a non-parametric test technique that was used to demonstrate linear patterns and is more efficient than the method using regression equations. If a time series possesses a linear trend, simple non-parametric method can be used to determine the true slope. In the sample of n pairs of data, SS test being used to calculate the trend slope (Eq. (6)).

(6) $$Q=\left(x_{j}-x_{k}\right)/\left(j-k\right)\;\text{for}\;i=1,\ldots ..,N$$

where, $x_{j}$ and $x_{k}$ are the data values at times j and k (j>k), correspondingly. If there is only a single observation in each time period, then (Eq. (7)) will be implied.

(7) N=n(n−1)/2

where, n is number of time periods. Eq. (7) will be implied if there are multiple observations in one or more time periods.

(8) N<n(n−1)/2

where n is the total number of observations. The SS estimator (median of slope) is calculated as in Eq. (9).

(9) $$Q_{med}=\left\{ \begin{array}{c} Q_{\left[N+1/2\right]},if\;N\;is\;odd\\ (Q_{\left[N/2\right]}+Q_{\left[N+2/2\right]}/2,if\;N\;is\;even \end{array}\right.$$

If the $Q_{med}$ shows a positive value, it signifies that the trend is an upward (increasing) trend, while a negative value shows a downward (decreasing) trend.

### 2.7 Rainfall variability analysis

Rainfall variability was determined in this study using Coefficient of Variation (CV), Precipitation Concentration Index (PCI), Standardized Precipitation Anomaly, and Rainfall Anomaly Index (RAI).

#### 2.7.1 Coefficient of variation (CV)

In order to calculate variability of rainfall, the CV value was determined (Eq. (10)). A high CV value signifies great variability, and vice versa (Table 2) [52].

(10) CV=σ/μ×100

where, CV is coefficient of variation; σ is standard deviation and μ is mean of rainfall.

**Table 2:** Degree variability of rainfall events classification.

CV values	Degree of rainfall variability
<20	Less
20–30	Moderate
>30	High

#### 2.7.2 Precipitation concentration index (PCI)

At different scales, the PCI value is applied to evaluate rainfall (annual or seasonal) heterogeneity (variety). Eq. (11) below was used to determine the PCI values, and Table 3 shows the classification [53].

(11) $${PCI}_{annual}=\sum_{i=1}^{12}p_{i}^{2}/\sum_{i}^{12}p_{i}\times 100$$

where, $p_{i}$ is rainfall amount of i ^th^ month.

**Table 3:** Precipitation Concentration Index (PCI) characteristics.

PCI values	Description
<10	Low rainfall concentration (uniform monthly distribution of rainfall)
11–15	Moderate rainfall concentration
16–20	High rainfall concentration
>21	Very high rainfall concentration

#### 2.7.3 Standardized precipitation anomaly

Instead, standardized precipitation anomaly (Eq. (12)) has been analyzed to determine the types of rainfall patterns, enables the identification of wet and dry years across the time series, and is applied to gauge the droughts frequency and severity (Table 4) [53].

(12) $$Z=\left(x_{i}-{\bar{x}}_{i}\right)/s$$

where Z is standardized precipitation anomaly; $x_{i}$ is annual rainfall for a certain year; ${\bar{x}}_{i}$ is long-term mean annual rainfall throughout the observation period and s is annual rainfall standard deviation over the observation period.

**Table 4:** Drought severity classes.

Value of Z	Drought severity classes
< −1.65	Extreme drought
−1.28 to −1.65	Severe drought
−0.84 to −1.28	Moderate drought
> −0.84	No drought

#### 2.7.4 Standardized precipitation index (SPI)

Standardized precipitation index (SPI) is a meteorological drought index recommended by World Meteorological Organization (WMO). The SPI was developed by Mckee in 1993 [54] where precipitation is the main influencing climatic factor [55]. The SPI is widely used drought index to characterize meteorological drought besides being used in frequency analysis and climate impact studies. The SPI assesses recorded precipitation by expressing it as a standardized deviation from a chosen probability distribution function, which represents the underlying precipitation data. The recorded precipitation data is commonly adjusted to either a gamma or a Pearson type III distribution, followed normal distribution transformation. Thus, the SPI values can be interpreted as the amount of standard deviations by which the observed anomaly differs from the long-term mean. It uses monthly precipitation aggregates at different time scales (1, 3, 6, 12, 18, and 24 months, etc.). Table 5 represent different degree of severity based on the SPI values [55].

**Table 5:** Degree of severity based on Standardized Precipitation Index (SPI).

SPI values	Category
≥2.00	Extreme wet
1.50–1.99	Severe wet
1.00–1.49	Moderate wet
−0.99–0.99	Near normal
−1.00 ∼ −1.49	Moderate drought
−2.00 ∼ −1.50	Severe drought
≤ - 2.00	Extreme drought

This study applied Climpact package in R-programming software to calculate SPI values at 4 different time scales (1, 3, 6 and 12).

The probability density function of the gamma distribution is defined as below (Eq. (13)):

(13) $$g\left(x\right)=1/\beta^{\alpha }\left(\alpha \right)x^{\alpha -1}e^{-x/\beta },\text{for}\;x>0$$

where x>0 is the amount of precipitation, a>0 and b>0 are the shape and scale parameters, respectively, and G(a) is the gamma function. Comprehensive information regarding gamma distribution can be obtained by referring to this studies [56,57]

To determine the distribution parameters, the values of a and b are estimated from the sample data using the maximum likelihood approximation [58], as the equation below (Eq. (14)):

(14) $$\hat{\propto }=1/4A\left(1+\sqrt{1+4A/3}\right),\hat{\beta }=\bar{x}/\hat{\alpha }\;\text{and}\;A=\ln\left(\bar{x}\right)-\sum \ln\left(x\right)/n$$

Where *x* is mean precipitation.

F or a given month and time scale, the cumulative probability G(x) of an observed amount of precipitation is given by:

(15) $$G\left(x\right)=\int_{0}^{x}g\left(x\right)dx=1/\beta^{\alpha }\gamma \left(\alpha \right)\int_{0}^{x}x^{\alpha -1}e^{-x/\beta }dx$$

The gamma distribution is not defined for *x* = *0*, and, the probability of zero precipitation q=P(x=0) being positive, the actual probability of non-exceedance H(x) is calculated as follows (Eq. (16)):

(16) H(x)=q+1(1−q)G(x)

Finally, the cumulative probability distribution H(x) is transformed into the standard normal distribution to produce the SPI values.

#### 2.7.5 Rainfall anomaly index (RAI)

By assigning magnitudes in the rainfall data to both positive and negative (severity) of rainfall anomalies, Van Rooy develops a ranking system [1]. It also reflected an index of significant technical simplicity as it only requires precipitation data [59]. The RAI concept used in previous study by Sanches [60] to evaluate precipitation variability across seven decades (1928–2009) in Alegrete and conclude that the RAI establishes to be an essential tool in analyzing precipitation data for the study area. Likewise, previous study by Santos [61] utilized the RAI in analyzing the climate of the Mamanguape River basin, where they identified three distinct regions within the river basin based on the precipitation patterns. The research outcomes suggested that RAI serves as an alternative tool for observing the precipitation patterns in the area.

The RAI is computed using the following equation (Eq. 17) and then classified into 9 regimes according to Table 6 [55].

(17) $$RAI=\pm 3\left(P-\bar{P}\right)/\left(E-\bar{P}\right)$$

where, P is measured rainfall; $\bar{P}$ is mean rainfall and $\bar{E}$ is mean of 10 extremes (mean of 10 highest rainfalls recorded in the period).

**Table 6:** Rainfall category based on standard ranges of Rainfall Anomaly Index (RAI).

RAI values	Rainfall category
>4.00	Extreme humid
2.0–4.0	Very humid
0.0–2.0	Humid
−2.0–0.0	Dry
−4.0 ∼ −2.0	Very dry
< −4.0	Extreme dry

## 3 Results

### 3.1 Homogeneity of rainfall

Homogeneity results (Table 7) show that six out of eight stations are labelled as Class A (Useful) which includes Kluang, Mersing, Senai, Pusat Pertanian Parit Botak, MARDI Pontian and MARDI Kluang. Meanwhile Pusat Pertanian Sungai Sudah and Chemara Layang-layang are labelled as Class B (Doubtful). These two stations are not the principal climate stations in Malaysia and might be less maintained and calibrated by the MetMalaysia staff [41]. Based on these results (Table 7), all the stations will be included for the trend analysis.

**Table 7:** Results of the Homogeneity test.

Class A (Useful)		Class B (Doubtful)		Class C (Suspect)
MY001	Kluang	MY005	PP Sungai Sudah	
MY002	Mersing	MY007	Chemara Layang-layang	
MY003	Senai			
MY004	PP Parit Botak			
MY006	MARDI Pontian			
MY008	MARDI Kluang			

### 3.2 Normality of rainfall

Based on the results of Shapiro–Wilk normality test (Table 8), it can be concluded that precipitation data for all the stations is not normally distributed. Therefore, non-parametric trend analysis will be used to determine precipitation trend of all the stations.

**Table 8:** Results of the Shapiro–Wilk normality test.

Var	MY001	MY002	MY003	MY004	MY005	MY006	MY007	MY008
Jan	0.31	0.25	0.08	**<0.001**^a^	0.21	0.41	0.51	0.09
Feb	**<0.001**^a^	0.35	0.31	0.23	**0.00**^a^	0.51	0.22	0.06
Mac	0.25	0.15	0.19	0.27	0.06	0.07	0.33	0.30
Apr	0.93	0.22	0.60	0.42	0.52	0.87	**<0.001**^a^	0.10
Mei	0.12	0.15	0.21	0.53	0.35	0.30	0.16	0.08
Jun	0.99	0.66	0.24	0.15	0.35	0.32	0.12	0.12
Jul	0.17	0.52	0.13	0.06	0.22	0.07	0.11	0.18
Aug	**0.02**^a^	0.28	0.41	0.51	0.12	0.49	0.41	0.53
Sep	0.30	0.83	0.24	0.32	0.42	0.25	0.35	0.24
Oct	0.08	0.50	**0.04**^a^	0.52	0.49	0.18	0.06	0.10
Nov	0.59	0.38	0.19	0.46	0.31	0.47	0.55	**0.04**^a^
Dec	0.10	**0.01**^a^	0.34	0.11	0.10	**0.02**^a^	0.22	0.32
Annual	0.06	0.89	0.58	0.46	0.43	0.94	0.31	0.28
SWM	0.25	0.80	0.47	0.74	0.94	0.65	0.48	0.50
NEM	0.20	0.60	0.92	0.44	0.11	0.41	0.62	0.64

Var = variable; SWM = Southwest monsoon; NEM = Northeast monsoon.

a p < 0.05; data is normally distributed.

### 3.3 Trend analysis

**Fig. 2:**
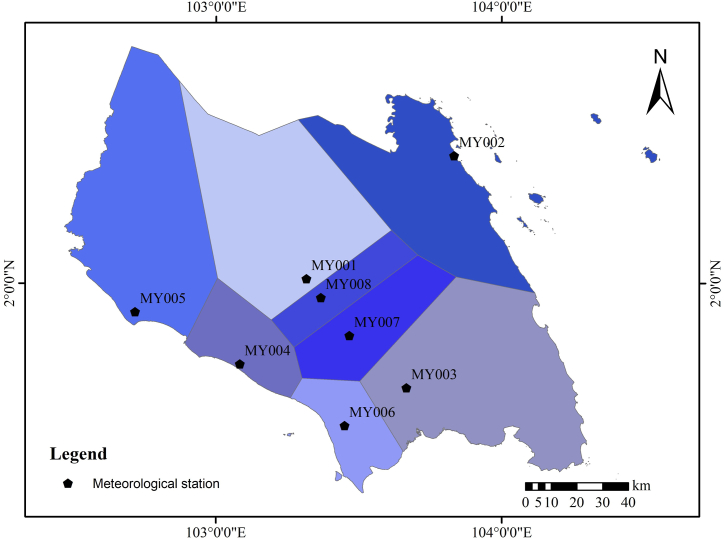
Location of meteorological station and rainfall distribution based on Thiessen polygon.

The rainfall trend time series data for monthly, annual, and seasonal means were examined using Man Kendall (MK) and Sen's slope (SS) test as the normality test results showed that those data is not normally distributed, where non-parametric test should be used for trend analysis. Rainfall data were calculated based on the assumptions made from the results from Thiessen polygon method (Fig. 2).

**Fig. 3:**
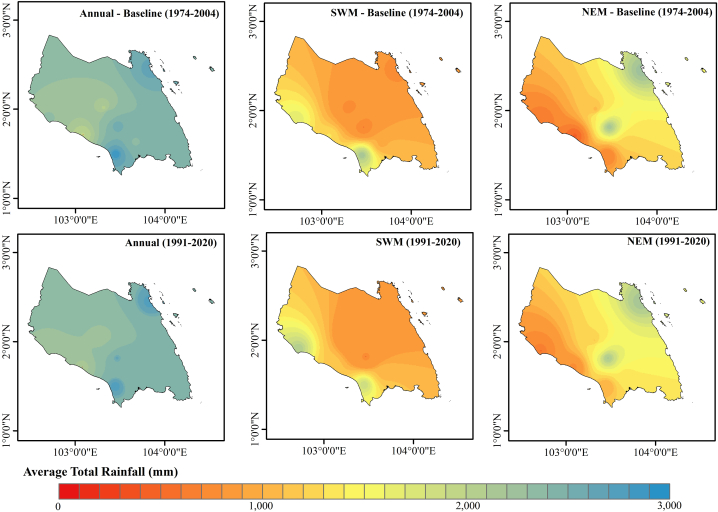
Average total rainfall for the period of 1991–2020.

**Fig. 4:**
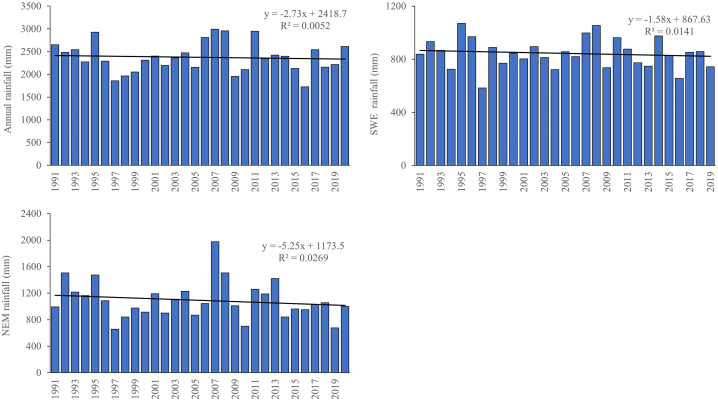
Comparison of Johor annual and seasonal monsoon rainfall pattern.

Table 9 displays MK statistics with p-values calculated at 5 % and 10 % significance levels. Parameters including Kendall's tau, S statistic, and Z statistic were employed in MK test to evaluate rainfall time series to determine either an upward or downward trend. Fig. 3 represents the average annual total annual and seasonal rainfall for the period of 1991–2021 and the based period of 1974–2004. The average annual rainfall for Johor throughout the study period (1991–2020) was 2376.45 mm, with SD of 332.41 mm and CF of 13.99 % (Table 9). A high SD value indicates that the measured rainfall values vary significantly. Based on Fig. 4, the lowest annual rainfall recorded was 1725.07 mm in 2016 (the driest year) and the highest was 2993.19 mm in 2007 (the wettest year). Minimum rainfall recorded in February (8.60 mm), while December recorded the maximum rainfall (787.48 mm). As the major rainy season in Malaysia, NEM season receives more rainfall (46.0 % of the annual rainfall) than the SWM season (36.0 % of the annual rainfall). This is concurrent with the study by Tang [62] that conclude Peninsular Malaysia experiences a rather hot and dry climate often with less rainfall and less cloud during SWM from late May to September. The monsoon weather system, which frequently happens in conjunction with the cold air outbreaks from Siberia, frequently causes catastrophic floods throughout NEM season in Peninsular Malaysia, particularly in Kelantan, Terengganu, Pahang, and East Johor as well as East Sarawak.

**Table 9:** Descriptive statistics and MK trend analysis of rainfall in Johor.

Month	Min	Max	Mean	SD	CV (%)	MK test	SS
Jan	28.00	645.19	217.69	130.15	59.78	−0.0161	−0.2615
Feb	8.60	407.91	105.10	84.85	80.73	−0.1724	−1.8574
Mar	86.83	431.22	191.67	86.57	45.16	−0.1218	−1.5896
Apr	120.38	321.80	215.41	49.03	22.76	**0.1862**^b^	1.5809
May	99.28	332.30	194.69	55.31	28.41	0.1126	1.0337
Jun	61.84	273.49	155.78	50.71	32.55	−0.0391	−0.3732
Jul	78.40	267.73	157.62	56.05	35.56	−0.1218	−1.2512
Aug	61.50	285.99	175.42	51.67	29.45	**−0.1770**^a^	−1.7268
Sep	49.59	244.44	172.87	36.81	21.29	**0.1770**^a^	0.9939
Oct	109.22	404.73	210.94	74.50	35.32	−0.0621	−0.5078
Nov	111.64	392.02	268.88	69.21	25.74	0.0667	0.6801
Dec	144.31	787.48	310.38	137.98	44.46	−0.1034	−1.2096
SWM	584.39	1215.16	856.38	130.17	15.20	−0.0115	−0.3472
NEM	658.25	1975.90	1092.14	282.06	25.83	−0.1218	−5.9443
Annual	1725.07	2993.19	2376.45	332.41	13.99	−0.0253	−2.2698

a statistically significant at 0.05 alpha level of significance.

b statistically significant at 0.1 alpha level of significance.

Percentage of coefficient values (CV) indicates the degree of rainfall variation (Table 9). The month of January, February, March, June, July, October, and December showed the most variability (CV > 30), followed by April, May, August, September. The NEM season showed intermediate variability (20 < CV < 30), while SWM season and annual data displayed the least variability (CV < 20). Based on Fig. 4, the ratio of changes is indicated by regression line slope; −2.73 mm/year for yearly rainfall, −1.58 mm/year for SWM season rainfall, and −5.25 mm/year for NEM season rainfall. After the flood that happened in Johor in the beginning of January 2007, the NEM season recorded the highest rate of rainfall reduction.

### 3.4 Rainfall anomaly

**Fig. 5:**
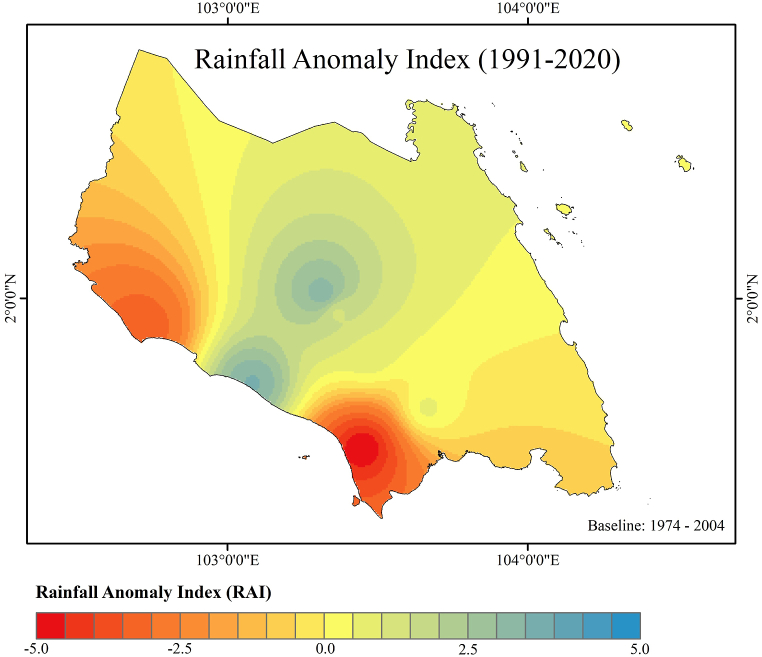
Rainfall anomaly index map (1991–2020).

**Fig. 6:**
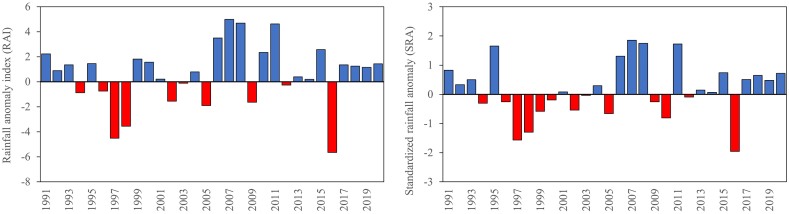
Comparison between rainfall anomaly index standardized precipitation anomaly.

In this study, rainfall anomaly index (RAI) and standard rainfall anomaly (SRA) were calculated to determine the rainfall anomaly pattern. The RAI was computed and classified based on annual rainfall. Fig. 5 represents the Johor RAI map for the time period of 1991–2020 with the baseline period of 1974–2004. Only some parts of Johor classified as extremely dry area with the RAI ≤ −4.0. Fig. 6 portrays the dry and rainy years based on RAI index values. Most of the year, RAI is positive (>0), whereas some years experience a dry year. The recorded RAI value falls within the range of 4.98 (extremely wet) to −5.65 (exteremely dry). The year of 1997, 1998 and 2016 experienced extremely dry conditions (RAI ≤ −4.0) in Johor while the year of 2006, 2007, 2008, 2010 and 2011 experienced extremely wet conditions (RAI ≥4.0).

Similarly, low values of SRA recorded for Johor (Fig. 6), ranges from +1.86 in 2007 to −1.96 in 2016 corresponding to severe drought periods incidents. Subsequent to the classification in Table 4, there were extreme drought occurrences in 2016, severe drought in 1997, and moderate drought in 1998. Fig. 6(a) potrayed exceptionally low values of standardized rainfall anomaly recorded for Johor, ranges from +1.86 in 2007 to −1.96 in 2016, corresponding to severe drought periods incidents. Subsequent to the classification in Table 4, there were extreme drought occurrences in 2016, severe drought in 1997, and moderate drought in 1998. This is concurrent with previous study by Mastura [63] reported that El-nino occurred in Malaysia during 1997, 1998 and 2016.

### 3.5 Standard precipitation index (SPI)

The SPI was calculated at four different time scales which include one month (SPI-1), three months (SPI-3), six months (SPI-6) and 12 months (SPI-12). The SPI-1 can be used to interpret short term drought conditions which can be related to soil moisture content that is very important for the agriculture sector. The SPI-3 provide seasonal estimation of rainfall, SPI-6 shows meso-scale trend in rainfall conditions and SPI-12 represents long-term rainfall period which can be vital to water supply management systems in terms of stream flows, reservoirs, and groundwater levels [64]. Table 10 represents the classification of rainfall regime based on SPI values. Mostly drought recorded classified as moderately dry and severely dry except for the year 1997, 1998. 2014, 2016 and 2019.

**Table 10:** Standard precipitation index classification.

SPI Category	Moderately dry		Severely dry		Extremely dry
SPI-1			1994	2014	1997
			2005	2015	1998
			2006	2018	2016
			2010	2019	
SPI-3	1992	2014			1998
	1997	2015			2019
	2004	2016			
	2006	2018			
SPI-6	2019	2016			
	2012	2015			
	1998	2018			
	2020				
SPI-12	1998	2016	2019		
	2002	2020			
	2015				

**Fig. 7:**
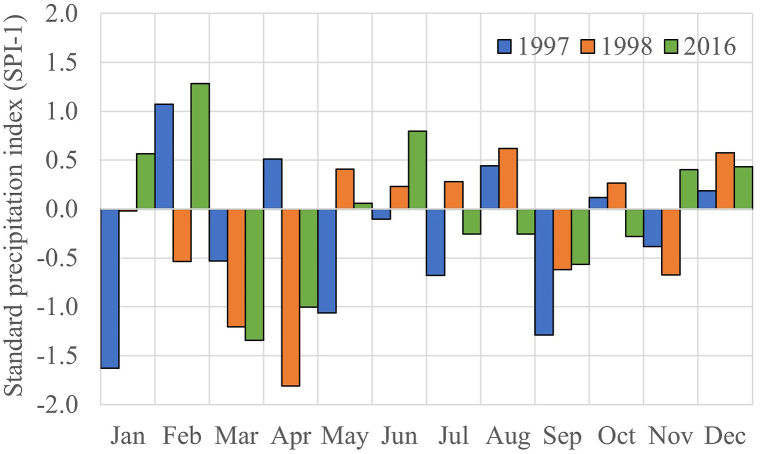
Standard precipitation index (SPI-1) for 1997, 1998 and 2016.

**Fig. 8:**
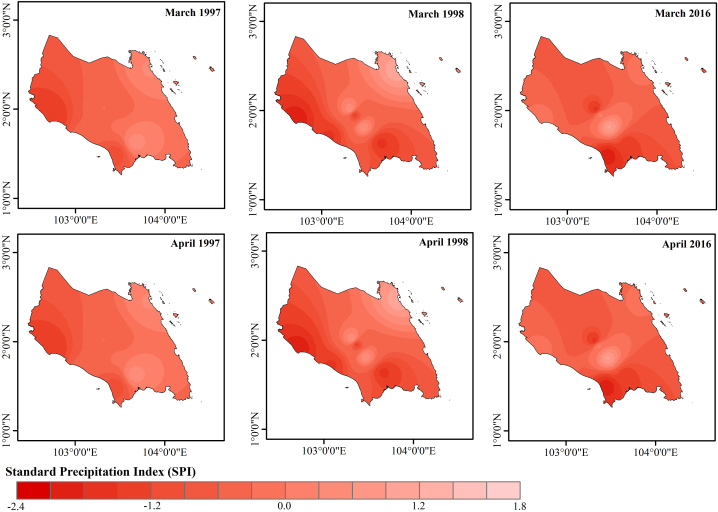
Drought severity map based on SP-1.

Therefore, based on the values of RAI, SRA and SPI, the years of 1997, 1998 and 2016 were selected to identify which month that drought occurred throughout the years (Fig. 7) based on SPI-1. Next, those months identified with their respective SPI values over the meteorological stations within the study area for further analysis to generate a Drought Severity Distribution Map of the study area as shown in Fig. 8.

### 3.6 Precipitation concentration index

**Fig. 9:**
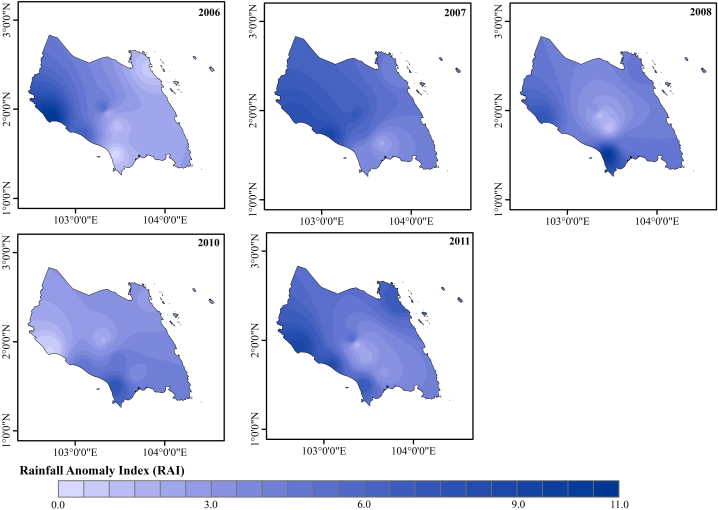
Rainfall anomaly index for the years 2006, 2007, 2008, 2010 and 2011.

The PCI is thought to provide information about long-term overall variability based on the amount of rainfall received, according to various earlier studies [65]. In this study, the PCI values were calculated based on annual rainfall data. The PCI values (Table 11) indicated the occurrence of low, moderate, and high concentration of rainfall. The mean PCI values for 1991 to 2020 is 11.39 which indicates a moderate concentration of rainfall where Johor experienced mostly low rainfall concentration (Table 11). Similar to RAI, Johor experienced a very high concentration of rainfall in 2006, 2007, 2008, 2010 and 2011 (Table 12). Therefore, these years were selected with their respective RAI values over the meteorological stations within the study area for further analysis to generate a RAI map of the study area as shown in Fig. 9.

**Table 11:** Precipitation concentration index (PCI) of Johor.

Index	Description	Number of years (1991–2020)
<10	Low rainfall concentration (almost uniform)	17
11–15	Moderate rainfall concentration	4
16–20	High rainfall concentration	9
≥21	Very high rainfall concentration	0
Mean PCI (1991–2020) = 11.39 (moderate concentration of rainfall)

**Table 12:** Precipitation concentration index (PCI) classification of Johor.

Precipitation concentration index category				
Low		Moderate	High	Very high
1992	2001	1991	2006	
1993	2002	2004	2007	
1994	2003	2014	2008	
1995	2005	2016	2010	
1996	2009		2011	
1997	2012		2015	
1998	2013		2018	
1999	2017		2019	
2000			2020

## 4 Discussions

Based on the results, it can be concluded that Johor do experience flood and drought throughout 1991 to 2020. Malaysia is generally known to experience dry conditions (wet conditions) during El Niño (La Niña) [66]. Corresponding to the research by Shafie [67], a storm that blows from Southeast China Ocean and West Pacific Ocean trigger heavy rainfall events that led to major floods in December 2006 and January 2007. Kota Tinggi district was hit severely from the storms that brought 287 mm and 338 mm of rain in four days logged in Bandar Kota Tinggi for the year 2006 and 2007, respectively. This fits with the flood event that took place in Johor throughout those years. Typhoon Utor, which passed over the central Philippines in December 2006 and caused floods in 2006 and 2007, also caused significant floods in Malaysia including Johor due to its prolonged tropical moisture together with high velocity monsoon winds [30].

In 2006, eighteen people lost their lives and the damage estimated around USD 489 million in these floods in Johor [68] whereas flood in 2007 had displaced 110,000 people, damaging an estimate of RM 0.35 billion worth of infrastructure and RM 2.4 billion of economics losses. An estimate of RM 84 million worth of agriculture produce was damaged or losses affecting 7000 farmers [69]. In 2008, floods devastated Johor once more, killing the 28 people and incurring a projected loss of USD 21.19 million [68]. Floods in 2011 were caused by heavy rainfall, which led to an increase in the water levels of Johor's three major rivers: Sungai Mengkibol, Sungai Muar, and Sungai Benut. This ultimately caused the rivers to overflow their banks, resulting in floods throughout the region [70]. In 2015, due to downpour with peak of 255 mm, some states in Peninsular Malaysia including Johor experienced floods. These floods are the worst in recent decades [30].

In Malaysia, extreme floods that occurred on December 15, 2014 to January 3, 2015 are the worst flood events in decades. During this event, most of the rivers in Johor, Kelantan, Pahang, Perak, and Terengganu reached dangerous levels. More than 200,000 people were affected and 21 people were killed due to this natural disaster [71]. Besides, an Institute for Medical Research (IMR) model shows that high rainfall is required for high transmission of dengue. There could be an increase in vector-borne disease – such as malaria and dengue fever – as changes in temperature will increase the availability of suitable breeding habitats for the vectors. As an example, between 1st August to August 20, 2016, a total of 71,590 dengue cases were reported in Malaysia with 162 deaths. The bulk of the cases were in the states of Selangor, Kelantan, Johor, and Kuala Lumpur [69]. In 2018, 2019, and 2020, the yearly NEM season in Johor brought heavy rainfall resulting in flood events [72,73]. A study from Shah which stated that south region of Malaysia had an intense rainfall for almost a week that led to flash flood in November 2019 and was one of the worst tragedies that have ever happened, especially in Johor as the number of flood victims rise rapidly [74]. Likewise, in June of 2020 (during the SWM season), heavy rains cause floods in Johor, forcing 1210 individuals from 288 households to move to 18 centres in Muar, Batu Pahat, Tangkak, Kluang, and Pontian districts [72].

Besides flood events, Johor also experienced drought. Thus, drought analysis is necessary for managing water, monitoring the dry events and mitigating the drought effects since drought episodes has been determined as a non-trivial issue and this analysis would be used in planning and managing the water resources systems for many decades [75]. Major El Niño events occurred in 1997, 1998, and 2016. Due to decreasing rainfall during this period, Malaysia has been experiencing extremely severe dry seasons [76,77]. During the El Niño event, Malaysia experienced an increase in temperature, which caused forest fires and ultimately contributed to the 1997–1998 haze crisis [78]. Forest fire in Indonesia at the same time worsened the impact of haze resulting mostly of Malaysia airspace covered by the haze from Indonesia thus forcing the Malaysian government to carry out the cloud seeding process to minimize the impact of haze [79]. The government at that time also obligated Malaysian citizen to wear mask to prevent dangerous disease [79]. El Niño returned in 2016, causing hydrological droughts in Malaysia. Timah Tasoh (Perlis), Bukit Kwong (Kelantan), Beris Padang Saga, Muda (Kedah), Bukit Merta (Perak), and Labong (Johor) dams' reserve water levels decreased to less than half of their capacities because of this [77]. Water shortage caused by hydrological drought reduces crop productivity which may lead to food shortage.

Various trends in rainfall are influenced by atmospheric circulation changes [5], which were triggered by human actions that increased the atmospheric concentration of greenhouse gases [80]. Moreover, changes in rainfall patterns are influenced by a location's topographical features [81], since weather patterns and climate circulation are influenced by land cover and land used based on local, regional, and global scale [52]. Anomalous dry and wet conditions occur in conjunction with El Niño and La Niña events, respectively. Tanggang [38] further revealed that the anomalous conditions over Malaysia and the Southeast Asia region during El Niño (La Niña) were modulated by regional atmosphere – ocean interaction in the presence of monsoonal background influences. The occurrence of disaster due extreme climate change such as floods and droughts, could impact damaging effect on the economy, social and psychology of the people affected. Thus, flood and drought events can cause series of destruction which humans should learn to interact well with the system so that both can thrive and survive in this dynamic and highly sensitive system accordingly [67] through adaptation and mitigation strategies.

The ability to adapt and mitigate to future changes in rainfall distribution pattern and water availability and flooding requires hydro-climatic projections at a local scale [41]. Therefore, further studies to investigate the past years rainfall trend and future projection is essential for upcoming research to gain a preliminary grasp of the likelihood of a changing climate. Rainfall data should be gathered from as many meteorological stations as possible to guarantee data quality and reduce errors to get a more accurate result.

## 5 Conclusions

Historical data recorded indicates that Johor experienced wet and dry years throughout the period of 1991–2020. These wet and dry years lead to extreme events such as flood and drought where La Nina and El Nino occurred respectively. Normality and homogeneity tests were done to ensure the quality of data. The MK trend analysis was used to determine rainfall trend as the data is not normally distributed, where results revealed a declining trend in monthly, annual, and seasonal rainfall although it is not statistically significant. The RAI, SRA and PCI were calculated to determine the rainfall variability and intensity. Results signify that Johor received huge amount of rainfall 2006, 2007, 2008, 2010 and 2011 which lead to major flood events. In the other hand, SPI was calculated to determined drought intensity where 1997, 1998 and 2016 represents the driest year which led to El Nino phenomenon where March and April happened to be the driest months. Flood and drought incidents have the potential to trigger a cascade of adverse effects. Thus, it is crucial for us to develop effective interactions with the system, enabling both parties to flourish and endure within this dynamic and highly sensitive environment. This can be achieved through the implementation of suitable adaptation and mitigation strategies. Consequently, the results of this study might be beneficial in providing data and evidence to the government, agencies, or other related parties involved in developing the appropriate adaptation and mitigation strategies to enhance adaptive capacity and decrease the effects of a changing climate.

## Data availability statement

Data will be made available on request.

## CRediT authorship contribution statement

**Shaidatul Azdawiyah Abdul Talib:** Writing – original draft, Visualization, Validation, Software, Resources, Methodology, Investigation, Formal analysis, Data curation, Conceptualization. **Wan Mohd Razi Idris:** Writing – review & editing, Supervision. **Liew Ju Neng:** Writing – review & editing, Supervision. **Tukimat Lihan:** Writing – review & editing, Supervision. **Muhammad Zamir Abdul Rasid:** Writing – review & editing.

## Declaration of competing interest

The authors declare that they have no known competing financial interests or personal relationships that could have appeared to influence the work reported in this paper.
